# In vivo recording of the circadian calcium rhythm in Prokineticin 2 neurons of the suprachiasmatic nucleus

**DOI:** 10.1038/s41598-023-44282-5

**Published:** 2023-10-09

**Authors:** Kaito Onodera, Yusuke Tsuno, Yuichi Hiraoka, Kohichi Tanaka, Takashi Maejima, Michihiro Mieda

**Affiliations:** 1 Department of Integrative Neurophysiology, Graduate School of Medical Sciences, Kanazawa University, Kanazawa, Ishikawa 920-8640 Japan; 2 Laboratory of Molecular Neuroscience, Medical Research Institute, Tokyo Medical and Dental University (TMDU), Tokyo, Japan

**Keywords:** Circadian mechanisms, Neurophysiology

## Abstract

Prokineticin 2 (Prok2) is a small protein expressed in a subpopulation of neurons in the suprachiasmatic nucleus (SCN), the primary circadian pacemaker in mammals. Prok2 has been implicated as a candidate output molecule from the SCN to control multiple circadian rhythms. Genetic manipulation specific to Prok2-producing neurons would be a powerful approach to understanding their function. Here, we report the generation of *Prok2-tTA* knock-in mice expressing the tetracycline transactivator (tTA) specifically in Prok2 neurons and an application of these mice to in vivo recording of Ca^2+^ rhythms in these neurons. First, the specific and efficient expression of tTA in Prok2 neurons was verified by crossing the mice with EGFP reporter mice. *Prok2-tTA* mice were then used to express a fluorescent Ca^2+^ sensor protein to record the circadian Ca^2+^ rhythm in SCN Prok2 neurons in vivo. Ca^2+^ in these cells showed clear circadian rhythms in both light–dark and constant dark conditions, with their peaks around midday. Notably, the hours of high Ca^2+^ nearly coincided with the rest period of the behavioral rhythm. These observations fit well with the predicted function of Prok2 neurons as a candidate output pathway of the SCN by suppressing locomotor activity during both daytime and subjective daytime.

## Introduction

The circadian clock in the suprachiasmatic nucleus (SCN) of the hypothalamus is the central circadian pacemaker in mammals, orchestrating multiple circadian biological rhythms in the organism^1^. The SCN contains approximately 20,000 cells, the majority of which are capable of generating circadian oscillations. Individual SCN cells possess intracellular molecular machinery (molecular clock) driven by the autoregulatory transcriptional/translational feedback loop (TTFL) of clock genes in cooperation with cytosolic signaling molecules, such as Ca^2+^ and cAMP^1^. Intriguingly, these molecular clocks are not unique to SCN cells and are common to peripheral cells^2^. Instead, intercellular communication between SCN cells is essential to generate a robust, coherent circadian rhythm as the central clock^1,3,4^.

The SCN is a heterogeneous structure composed of multiple types of GABAergic neurons and glial cells^1,5^. Co-expressing neuropeptides characterize several subtypes of SCN GABAergic neurons. For example, vasoactive intestinal peptide (VIP)-positive neurons in the ventral core region and arginine vasopressin (AVP)-positive neurons in the dorsal shell region are two representative neuron types in the SCN^6^. VIP is known to be the most critical contributor to the synchronization among SCN neurons and is also involved in the photoentrainment to regulate the phase of circadian rhythms according to the external light–dark (LD) cycle^7–13^. On the other hand, rhythmic clock gene expression is most prominent in the SCN shell, which mostly overlaps with the area containing AVP neurons^14^. Consistently, AVP neurons and other shell cells have been implicated in the generation and period-setting of the circadian rhythm by the SCN network^15–19^.

Prokineticin 2 (Prok2) belongs to a pair of unique cysteine-rich secreted proteins and has been implicated in regulating diverse biological processes, including olfactory bulb neurogenesis, pleasant touch sensation, and inflammation^20–25^. In particular, several lines of evidence support its candidate role as an output molecule of the SCN central clock to control the circadian behavioral rhythm. Prok2-positive neurons are distributed throughout the SCN in both the shell and the core^26–28^. *Prok2* mRNA expression is driven by the TTFL and thus exhibits robust circadian rhythm in the mouse SCN, peaking around zeitgeber time (ZT)/circadian time (CT) 4^27^. Its expression is also dependent on neuronal activity^29^. In addition, the receptor for Prok2 (Prokr2) is abundantly expressed in primary target nuclei of the SCN output pathway^27,30^. Furthermore, central administration of Prok2 suppresses nocturnal locomotor activity in rats^27^. Thus, Prok2 is likely released during the (subjective) day and inhibits locomotor activity to generate the circadian behavioral rhythm in nocturnal rodents. Correspondingly, *Prok2*- and *Prokr2*-deficient mice have attenuated circadian rhythms of behavior and other physiological parameters without affecting TTFL in their SCN^31,32^.

To elucidate how Prok2 neurons regulate circadian rhythms, genetic manipulations specific to these neurons for the recording and artificial manipulation of their activity would be a powerful approach. Here, we have established a *Prok2-tTA* knock-in mouse line that allows genetic manipulation of Prok2 neurons via the Tet system. The Tet system is a widely used genetic tool in which the tetracycline transactivator (tTA) binds the tetracycline-responsive element (TRE) and activates the gene downstream of the TRE^33,34^. In addition, we used this mouse tool to record the circadian rhythm of intracellular Ca^2+^ concentration ([Ca^2+^]_i_) in SCN Prok2 neurons in vivo and revealed its temporal relationship to the behavioral rhythm. In neonatal explants, SCN cells show robust daily [Ca^2+^]_i_ rhythms that depend on the TTFL, and the TTFL is also regulated by cytosolic Ca^2+^^35,36^. Moreover, differential [Ca^2+^]_i_ rhythms in different SCN neuron subtypes have been reported both in slices and in vivo^5,19,37–39^. Therefore, recording [Ca^2+^]_i_ rhythm from Prok2 neurons would be a first step towards understanding their precise role in circadian time-keeping.

## Results

### Generation of *Prok2-tTA* knock-in mice

**Figure 1:**
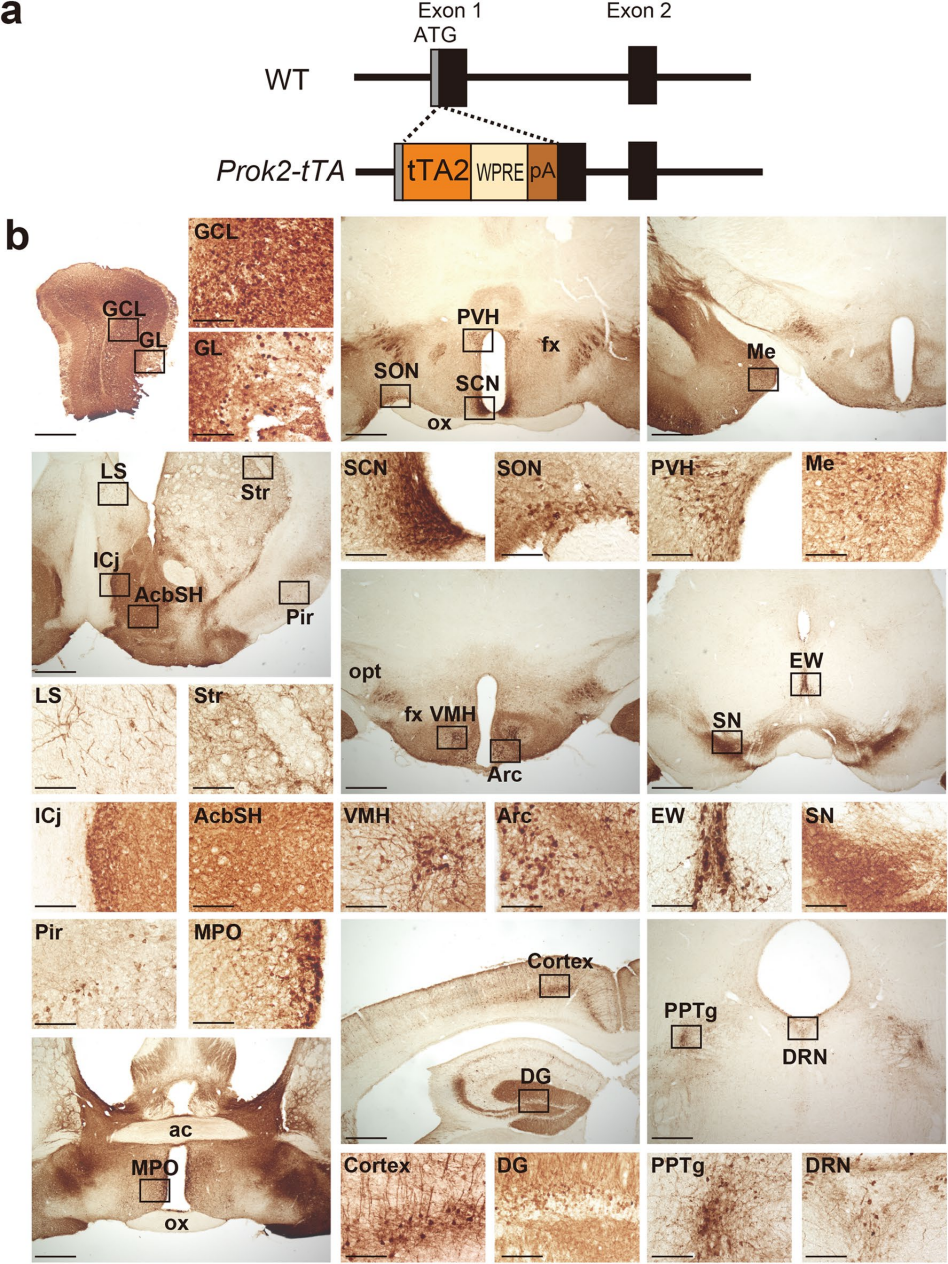
Generation of *Prok2-tTA* mice. (**a**) Targeting strategy for generating *Prok2-tTA* mice. (**b**) Coronal brain sections prepared from colchicine-pretreated *Prok2-tTA; Actb-tetO-EGFP* mice were immunostained for EGFP in brown. tTA-mediated EGFP expression mostly recapitulates reported *Prok2* expression in *Prok2-tTA* mice crossed with *Actb-tetO-EGFP* reporter mice. Regions containing EGFP + cell bodies are shown in panels with higher magnification. Some stained regions, such as the substantia nigra (SN), contain only EGFP + fibers and few EGFP + cell bodies. Scale bars, 100 µm or 500 µm for high or low magnification images. ac, anterior commissure; AcbSH, nucleus accumbens shell; Arc, arcuate nucleus; Cortex, cerebral cortex; DG, dentate gyrus; fx, fornix; DRN, dorsal raphe nucleus; EW, Edinger-Westphal nucleus; GCL, granule cell layer; GL, glomerular layer; ICj, islands of Calleja; LS, lateral septum; Me, medial amygdala; MPO, medial preoptic area; opt, optic tract; ox, optic chiasm; PAG, periaqueductal gray; Pir, piriform cortex; PPTg, pedunculopontine tegmental nucleus; PVH, paraventricular hypothalamic nucleus; SCN, suprachiasmatic nucleus; SN, substantia nigra; SON, supraoptic nucleus; Str, striatum; VMH, ventromedial hypothalamic nucleus.

To elucidate the function of Prok2 neurons, genetic manipulations specific to these neurons would be useful. For this purpose, we generated knock-in mice expressing tTA2^40^ specifically in Prok2 neurons. To do so, we employed the CRISPR/Cas9-mediated homologous recombination to target the *Prok2* gene of the mouse genome and inserted a *tTA2-WPRE-polyA* cassette near the *Prok2* gene start codon in its exon 1 in C57BL/6 J mice (*Prok2-tTA*) (Fig. 1a). To localize tTA2 activity, we crossed *Prok2-tTA* mice with *Actb-tetO-EGFP* reporter mice, which express EGFP in the presence of tTA^41,42^. EGFP + cells were observed in several brain regions that were reported to express *Prok2*^28,30,43^, including the olfactory bulb, nucleus accumbens, lateral septum, islands of Calleja, medial preoptic area, SCN, paraventricular hypothalamic nucleus, arcuate nucleus, and the Edinger-Westphal nucleus (Fig. 1b). In addition, we found EGFP + cells in the ventromedial hypothalamic nucleus and the pedunculopontine tegmental nucleus. Furthermore, EGFP + cells were distributed sparsely in the cerebral cortex, striatum, and hippocampus. EGFP + cells in the regions where *Prok2* expression has not been reported might result simply from ectopic expression or possibly from better sensitivity due to the use of the Tet system and WPRE (woodchuck hepatitis virus posttranscriptional regulatory element).

**Figure 2:**
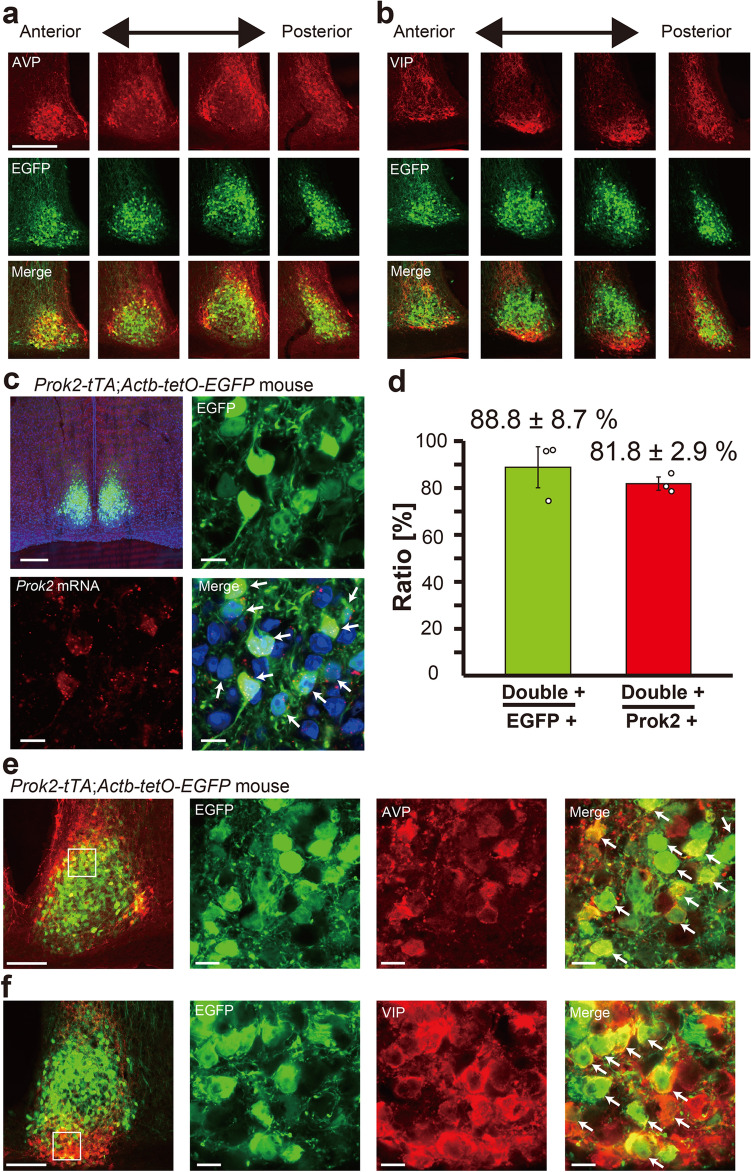
*Prok2-tTA*-mediated EGFP expression in the SCN. (**a**, **b**) Coronal brain sections prepared from *Prok2-tTA; Actb-tetO-EGFP* mice were immunostained in red for AVP (**a**) or VIP (**b**). For fluorescent immunostaining, mice were pretreated with intracerebroventricular injections of colchicine for 48 h before transcardial perfusion of fixative. (**c**) In situ HCR was performed to detect *Prok2* mRNA (red dots) on coronal brain sections prepared from *Prok2-tTA; Actb-tetO-EGFP* mice. Sections were counterstained with DAPI (blue). (**d**) Proportions of *Prok2* + :EGFP + cells to EGFP + cells (Double + /EGFP +) or *Prok2* + cells (Double + /*Prok2* +). n = 3. Double positive cells are indicated by white arrows. Bars indicate mean values ± SEM. Open circles are individual values. (**e**, **f**) High magnification images of (**a**) or (**b**). Scale bars, 200 μm (**a**–**c**) or 100 μm (**e**, **f**) for low magnification images; 10 μm for high magnification images (**c**–**f**). Double positive cells are indicated by white arrows.

As expected, the SCN was one of the brain regions containing many EGFP + cells. EGFP + cells were distributed throughout the SCN, from anterior to posterior, in both the shell and the core of the SCN (Fig. 2a,b). Within the SCN, EGFP expression was almost completely colocalized with *Prok2* mRNA expression detected by in situ hybridization chain reaction (HCR) (88.8 ± 8.7% of EGFP + cells were also *Prok2* + , 81.8 ± 2.9% of *Prok2* + cells were also EGFP + , n = 3) (Fig. 2c,d). Reportedly, *Prok2* mRNA expression partially overlaps with *Avp* and *Vip* mRNA expression in the SCN^26,28,44–47^. Indeed, ~ 20% of EGFP + cells showed AVP immunoreactivity (19.5 ± 1.3%, n = 5), whereas ~ 30% of AVP + cells were also EGFP + (31.4 ± 1.1%, n = 5) (Fig. 2a,e). Similarly, ~ 5% of EGFP + cells showed VIP immunoreactivity (5.7 ± 0.4%, n = 5), whereas ~ 20% of VIP + cells were also EGFP + (20.6 ± 1.9%, n = 5) (Fig. 2b,f). Most Prok2 neurons were distributed dorsally to VIP neurons, surrounded medially, laterally, and dorsally by AVP neurons. Thus, tTA2 expression occurred specifically and efficiently in Prok2 neurons within the SCN, confirming that *Prok2-tTA* mice are a valuable tool for Prok2-neuron-specific genetic manipulations.

### Heterozygous *Prok2-tTA* mice show normal circadian behavior rhythm

The *Prok2-tTA* allele should be equivalent to a *Prok2* knockout allele because the *Prok2* coding sequence was interrupted by a *tTA2-WPRE-polyA* sequence. In addition, previous studies reported attenuated circadian rhythms in *Prok2*- and *Prokr2*-deficient mice. Therefore, we tried to obtain homozygous *Prok2-tTA* mice by intercrossing heterozygous mice. However, no homozygous mice grew up to weaning, whereas 5 wildtype and 18 heterozygous littermates did, suggesting postnatal lethality of homozygous mice. This result was consistent with previous observations of *Prok2*- and *Prokr2*-deficient mice that their postnatal survival rates drastically dropped after backcrossing to C57BL/6 for 6 ~ 7 generations^31,32^.

To confirm that the *Prok2-tTA* allele causes no overt effect on circadian behavior, we next recorded the daily rhythms of spontaneous locomotor activity of heterozygous *Prok2-tTA* mice (Fig. S1). These mice demonstrated clear circadian behavioral rhythm in both light–dark (LD) and constant dark (DD) conditions, comparable to those reported for control mice in similar genetic backgrounds and recording conditions^15,16,39^.

### In vivo recording of the circadian Ca^2+^ rhythm in SCN Prok2 neurons

**Figure 3:**
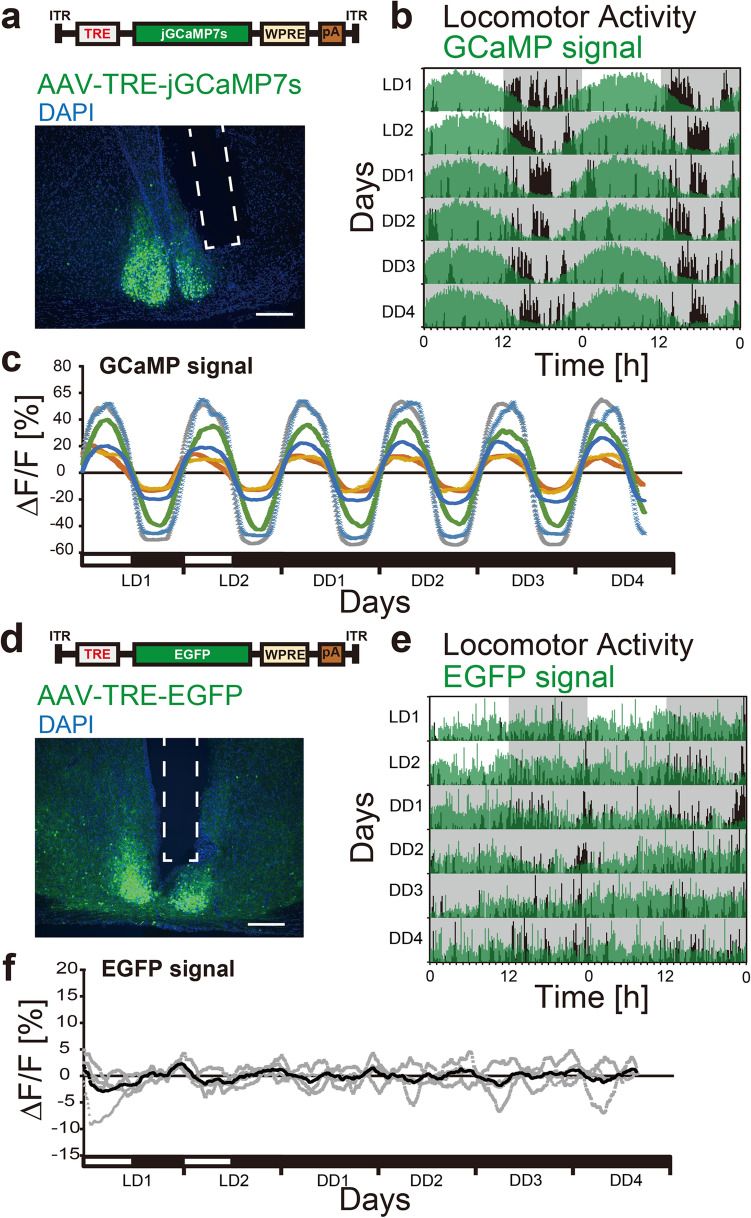
In vivo circadian [Ca^2+^]_i_ rhythm in SCN Prok2 neurons in freely moving mice. (**a**, **d**) jGCaMP7s (**a**) or EGFP (**d**) was expressed in SCN Prok2 neurons by focal injection of tTA-dependent AAV vectors in *Prok2-tTA* mice. Representative coronal sections with jGCaMP7s or EGFP expression in the SCN and estimated implanted optical fiber positions (white dotted square) are shown. Green, jGCaMP7s or EGFP; blue, DAPI. Scale bar, 200 μm. (**b**, **e**) Representative plots of the in vivo jGCaMP7s (**b**) or EGFP (**e**) signal of SCN Prok2 neurons (green) overlaid with locomotor activity (black) in actograms. Mice were initially housed in LD (LD1, 2) and then in DD (DD1-4). The dark periods are represented as gray-shaded areas. (**c**, **f**) Continuous recordings of jGCaMP7s (**c**) or EGFP (**f**) fluorescence from SCN Prok2 neurons for 6 days (2 in LD, 4 in DD). Detrended, smoothened data of individual mice are shown. In (**c**), each color indicates a different animal. In (**f**), the black line is the average of individual EGFP signals in gray. n = 6 for jGCaMP7s, n = 4 for EGFP.

Prok2 has been implicated as an output molecule of the central circadian clock of the SCN, which is released during the (subjective) day to suppress locomotor activity in mice^27^. For Prok2 to play this role, Prok2 neurons should also be active during the (subjective) day. To directly test this possibility, we next recorded the [Ca^2+^]_i_ rhythm in SCN Prok2 neurons in vivo by fiber photometry^39,48^ while monitoring the locomotor activity rhythm. To do this, the fluorescent Ca^2+^ indicator jGCaMP7s^49^ was expressed specifically in these neurons by focal injection of a tTA-dependent AAV vector (Fig. 3a).

In LD, when plotted on the actogram, a daily [Ca^2+^]_i_ rhythm was observed in SCN Prok2 neurons, higher during the light phase and lower during the dark phase (Fig. 3b,c). Such [Ca^2+^]_i_ rhythms persisted in DD, confirming that the observed rhythms were truly circadian and not driven by the external LD cycle. Importantly, our fiber photometry method did not detect a significant circadian oscillation of fluorescence when control EGFP was expressed in the SCN Prok2 neurons (Fig. 3d–f). Thus, our measurements of jGCaMP7s fluorescence were likely to reflect [Ca^2+^]_i_ in Prok2 neurons correctly.

**Figure 4:**
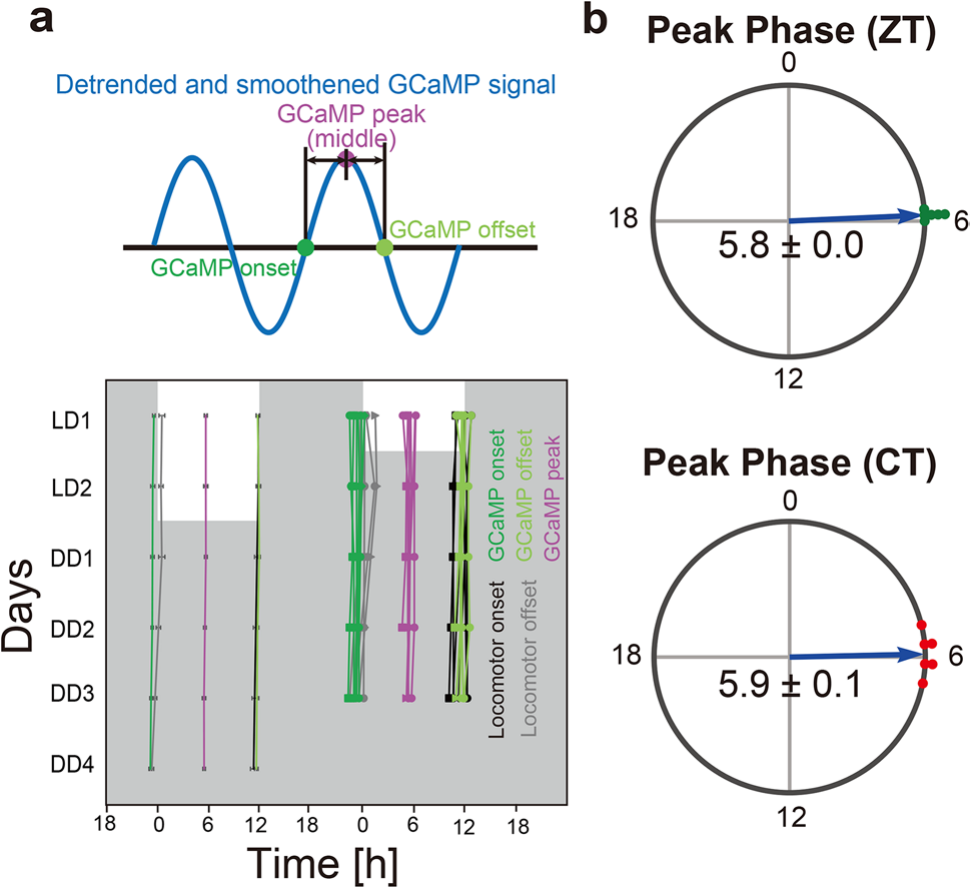
The phase relationship of the [Ca^2+^]_i_ rhythm in SCN Prok2 neurons and behavior rhythm. (**a**) Plots of locomotor activity onset (black), activity offset (gray), GCaMP onset (green), GCaMP offset (light green), and GCaMP peak (magenta) of mean ± SEM (left column) and individual mice data (right column). Identical marker shapes indicate data from the same animal. (**b**) Peak phases of the GCaMP fluorescence rhythm in LD (top) or DD (bottom) were shown as Rayleigh plots. Individual dots indicate the peak phases of each mouse. Values are mean ± SEM. n = 6.

For quantitative analyses, we defined the peak phase and period of the [Ca^2+^]_i_ rhythms, as well as the onset and offset of the hours of high [Ca^2+^]_i_ (Fig. 4a top)^19^. For this purpose, the data were detrended to remove the gradual signal decrease over the recording days and then smoothened to remove fast signal fluctuations within hours. Daily Ca^2+^ onset and offset were defined as the times when the value crossed 0 upward (i.e., [Ca^2+^]_i_ rising) and downward (i.e., [Ca^2+^]_i_ falling), respectively. The midpoints of Ca^2+^ onset and offset were defined as the peak phases, and the intervals between two adjacent peaks were defined as the periods. We considered these definitions are more appropriate than other methods, such as sine curve fitting, because the waveforms of [Ca^2+^]_i_ rhythms appeared to deviate from the typical sinusoidal curve and to be noisy with multiple small peaks within the hours of high [Ca^2+^]_i_ (Fig. 3c).

The peak of the [Ca^2+^]_i_ rhythm in SCN Prok2 neurons was in the middle of the day in LD (ZT5.8 ± 0.0) and the subjective day in DD (CT5.9 ± 0.1) (Fig. 4b). Its period in LD (24.0 ± 0.1 h) was equal to the 24 h LD cycle, and that in DD (23.9 ± 0.0 h) was comparable to the behavioral free-running period (23.8 ± 0.1 h). Intriguingly, the daily onset and offset of Ca^2+^ almost coincided with the offset and onset of the behavioral activity period, respectively (Fig. 4a bottom and Fig. S2).

## Discussion

In this study, we generated *Prok2-tTA* knock-in mice in which the tTA2-coding sequence was introduced into the endogenous *Prok2* locus. The expression of tTA was primarily restricted to the brain regions reported to express *Prok2* mRNA. As expected, the SCN contained many tTA-positive cells in both the shell and the core. Furthermore, the expression of tTA was highly specific for Prok2 neurons in the SCN. Therefore, in combination with transgenic mice or viral vectors with TRE-mediated transgene expression, *Prok2-tTA* mice can express any protein specifically in Prok2 neurons. In addition, many Cre driver mice specific for a particular type of SCN neurons are currently available, such as *Avp-ires-Cre*^50^, *Avp-Cre*^16^, *Vip-ires-Cre*^51^, *Nms-Cre*^52^, *Grp-Cre*^53^, *Drd1a-Cre*^54^, and *Vipr2-Cre*^38^. Therefore, by crossing one of them with *Prok2-tTA* mice, the double transgenic mice would allow us to simultaneously apply different genetic manipulations to Prok2 and another type of SCN neurons via the Cre/loxP and Tet systems. Such a dual-targeting strategy would allow us to directly study the interactions between Prok2 neurons and other SCN neurons, which would be a powerful tool for studying the SCN, a small but complex neuronal network composed of many types of neurons, including Prok2 neurons^1^.

A previous study reported co-localization of *Prok2* and *Avp* or *Vip* mRNA in the rat SCN: approximately 50.2% of *Avp* + cells expressed *Prok2* whereas 29.4% of *Prok2* + cells expressed *Avp*; approximately 41.6% of *Vip* + cells expressed *Prok2* whereas 21.8% of *Prok2* + cells expressed *Vip*^26^. We also observed a similar partial overlap of *Prok2-tTA* expression with AVP or VIP peptide. The slight difference in the proportions of double-positive cells may be due to differences in species and methods used to detect expression (i.e., in situ hybridization vs. immunostaining). Although we pretreated the mice with colchicine to reduce neuropeptide transport to the nerve terminals, thereby facilitating cell type identification, we sometimes encountered difficulties distinguishing immunoreactive cell bodies from nerve terminals, which could result in somewhat ambiguous cell counts. Intriguingly, Prok2 neurons were predominantly distributed in the central part of the coronal sections of the middle SCN, surrounded ventrally by VIP neurons and dorsally, medially, and laterally by AVP neurons. Since AVP and VIP neurons have been suggested to play different roles in the circadian pacemaking of the SCN network, it would be interesting to investigate in the future whether Prok2 also has different functions between Prok2 + /AVP + , Prok2 + /VIP + , and Prok2 + /AVP − /VIP − neurons.

Using *Prok2-tTA* mice, we successfully recorded the [Ca^2+^]_i_ rhythm in SCN Prok2 neurons in vivo. Its peak phase was around the midday (CT5.9 ± 0.1) and almost the same as that of VIP neurons (CT5.6 ± 0.2), but later than that of AVP neurons (CT3.2 ± 0.7)^19^. It has been reported that the rhythm of *Prok2* mRNA expression in the SCN peaks around CT4^26,27^. Therefore, the [Ca^2+^]_i_ rhythm may be slightly delayed compared to the mRNA rhythm. Because protein synthesis and maturation often lag minutes to hours behind mRNA transcription, the Prok2-neuronal [Ca^2+^]_i_ rhythm seems temporally organized according to *Prok2* expression. Notably, the rise and fall of Prok2-neuronal [Ca^2+^]_i_ mostly delineated the rest period of the behavioral rhythm. These observations fit well with the function of Prok2 as a candidate SCN output molecule released during the (subjective) day to suppress locomotor activity^27^.

## Methods

### Ethics statements

All experiments were performed in accordance with the Japanese Neuroscience Society and Kanazawa University guidelines for laboratory animal care and use. Experimental protocols were approved by the Animal Care and Use Committee and Gene Recombination Experiment Safety Committee of Kanazawa University and Tokyo Medical and Dental University. The study was carried out in compliance with the ARRIVE guidelines.

### Animals

To generate *Prok2-tTA* mice, we inserted a *tTA2-WPRE-polyA* cassette 25 bp downstream to the start codon of *Prok2* gene in its first exon by the CRISPR/Cas9-mediated targeting strategy as described previously^41^ (Fig. 1a). The donor DNA was synthesized, containing *tTA2* cDNA^40^, woodchuck hepatitis virus posttranscriptional regulatory element (WPRE), polyA signal derived from human growth hormone gene, and 1.5 kb sequences of the mouse *Prok2* gene (NCBI Gene: 50501) 5’ and 3’ to the insertion site. Endogenous initiation codon was inactivated by an A to T mutation. One-cell stage zygotes were obtained by mating C57BL6/J males and females (CLEA Japan). *Prok2*-crRNA (5′- CAGCAGAAGUAGCAGUAGCGguuuuagagcuaugcuguuuug-3′) and tracrRNA (5′-AAACAGCAUAGCAAGUUAAAAUAAGGCUAGUCCGUUAUCAACUUGAAAAAGUGGCACCGAGUCGGUGCU-3′) were chemically synthesized and purified by high performance liquid chromatography (Fasmac). A mixture of recombinant Cas9 proteins (NEB), *Prok2*-crRNA, tracrRNA, and *pProk2-tTA2-WPRE-polyA* targeting vector were injected into pronuclei of one-cell stage zygotes using a micromanipulator/microscope (Leica) and injector (Eppendorf). Embryos were then washed and cultured for over an hour in KSOM medium (ARK resource) and transferred into pseudopregnant ICR female mice (CLEA Japan). Presence of the knock-in allele was verified by PCR using tail genomic DNA. One F0 founder mouse was obtained, backcrossed at least twice with C57BL/6 J, and then used for the experiments in heterozygous condition. To evaluate the specific expression of tTA2, *Prok2-tTA* mice were crossed to *Actb-tetO-EGFP* reporter mice^41,42^. All mice were maintained under a strict 12 h light/12 h dark cycle in a temperature- and humidity-controlled room and fed ad libitum.

### Histological study

*Prok2-tTA; Actb-tetO-EGFP* mice were sacrificed around ZT4 ~ 5 by transcardial perfusion of PBS followed by 4% paraformaldehyde fixative. Serial coronal brain slices (30 µm thick) were prepared using a cryostat (CM1860, Leica) and collected in four series. One of these was further subjected to in situ hybridization chain reaction (HCR) or immunostaining.

In situ HCR for *Prok2* mRNA was performed using in HCR v3.0^55^ (Molecular Instruments). Prior to pre-hybridization, sections were pretreated as previously described for in situ hybridization, with proteinase K treatment replaced by 1% sodium borohydride to avoid digestion of the EGFP protein^56^. Pre-hybridization and subsequent procedures were performed essentially according to the protocol for fixed frozen tissue sections provided by Molecular Instruments (http://molecularinstruments.org), except that the sections were floated in solution in a 2 mL microcentrifuge tube. The probe set for *Prok2* was designed and synthesized by Molecular Instruments (lot number: PRJ347) and used in combination with HCR Amplifier B1 labeled with Alexa Fluor594 (Molecular Instruments). EGFP expression was detected by its native fluorescence.

Immunostaining was performed as previously described^16^. *Prok2-tTA; Actb-tetO-EGFP* mice were pretreated with intracerebroventricular colchicine injections (40 µg in 1 µl saline) for 48 h prior to perfusion fixation to accumulate peptides in the cell bodies. The antibodies used were: rabbit anti-AVP (Millipore, 1:4000); rabbit anti-VIP (Immunostar, 1:1000); and Alexa 488-conjugated goat anti-rabbit IgG (Molecular Probes, 1:1000). For Fig. 1b, EGFP was immunostained by Avidin/Biotin Method with rabbit anti-GFP antibody (Thermo Fisher Scientific, 1:1000), biotinylated goat anti-rabbit IgG antibody (Vector Lab, 1:1000), VECTASTAIN Elite ABC-HRP Kit (PK6100, Vector Lab), and DAB Substrate kit (SK4100, Vector Lab).

Stained sections were mounted on slide glasses with mounting medium (VECTASHIELD HardSet with DAPI, Vector Labs for fluorescence; Entellan New, Merck for DAB staining) and observed via epifluorescence or bright-field microscopy (KEYENCE, BZ-9000E) and laser-confocal microscopy (Olympus, FluoView FV10i).

### Behavioral analyses

3 male and 1 female heterozygous mice, aged 12 to 40 weeks, were housed individually in a cage placed in a light-tight chamber (light intensity was approximately 100 lx). Spontaneous locomotor activity (home-cage activity) was monitored by infrared motion sensors (Melquest) in 1-min bins as described previously^16^. Actogram, activity profile, and χ^2^ periodogram analyses were performed via ClockLab (Actimetrics). The free-running period was measured by periodogram for days 8–21 in DD. The activity time was calculated from the daily activity profile (average pattern of activity) of the same 14 days using the mean activity level as a threshold for detecting the onset and the offset of activity time^16^.

### AAV vectors

The AAV-2 ITR-containing plasmid *pAAV-TRE-EGFP* (Addgene plasmid #89875^57^, a gift from Dr. Hyungbae Kwon) was obtained from Addgene. *pAAV-TRE-jGCaMP7s* was constructed by replacing a *ChrimsonR-mCherry* EcoRI-HindIII fragment of *pAAV-TRE-ChrimsonR-mCherry* (Addgene plasmid #92207^58^, a gift from Dr. Alice Ting) with a EcoRI-HindIII fragment containing *jGCaMP7s* derived from a similar plasmid described previously^42^. Recombinant AAV vectors (AAV2-rh10) were produced by a triple-transfection, helper-free method and purified as previously described^16^. The titers of recombinant AAV vectors were determined by quantitative PCR: AAV-*TRE-EGFP*, 5.7 × 10^11^; AAV-*TRE-jGCaMP7s*, 5.8 × 10^12^ genome copies/ml.

### In vivo fiber photometry

We used four and six heterozygous *Prok2-tTA* mice for EGFP (control) and jGCaMP7s recordings, respectively. They were 6–11 months old and included both males and females. Focal injection of AAV vectors and optic fiber implantation were performed as previously described^39^. We injected 1.0 µL of the virus (AAV-*TRE-EGFP* or AAV-*TRE-jGCaMP7s*) into the right SCN (posterior: 0.5 mm, lateral: 0.25 mm, depth: 5.7 mm from the bregma) with a 33 G Hamilton Syringe (1701RN Neuros Syringe, Hamilton) to label Prok2 neurons. We then placed an implantable optical fiber (400 µm core, N.A. 0.39, 6 mm, ferrule 2.5 mm, FT400EMT-CANNULA, Thorlabs) over the SCN (posterior: 0.2 mm, lateral: 0.2 mm, depth: 5.3 mm from the bregma) with dental cement (Super-bond C&B, Sun Medical). The dental cement was colored black. Mice were used for experiments 2–4 weeks after the virus injection and optical fiber implantation.

A fiber photometry system (COME2-FTR, Lucir) was used to record the calcium signal of Prok2 neurons in freely moving mice^19,39,48^. A Fiber-Coupled LED (M470F3, Thorlabs) with LED Driver (LEDD1B, Thorlabs) was used as the excitation blue light source. The light was reflected by a dichroic mirror (495 nm), passed through an excitation bandpass filter (472/30 nm), and then delivered to the animal via a custom-made patch cord (400 um core, N.A. 0.39, ferrule 2.5 mm, length 50 cm, COME2-FTR/MF-F400, Lucir) and the implanted optical fiber. We detected the jGCaMP7s fluorescence signal with a photomultiplier through the same optical fibers and an emission bandpass filter (520/36 nm); furthermore, we recorded the signal using Power Lab (AD Instruments) with Lab Chart 8 software (AD Instruments). The intensity of the blue excitation light was 15–20 µW at the tip of the patch cord on the animal side. We recorded the signal for 30 s every 10 min to reduce photobleaching. During the recording, the mouse was housed in a 12-h light–dark cycle for two days (LD condition) and then transferred to continuous darkness for approximately four days (DD condition) in a custom-made acrylic cage surrounded by a sound-attenuating chamber. A swivel joint for the patch cord was stopped during the recording to prevent artificial baseline fluctuations. The animal's locomotor activity was monitored with an infrared sensor (Supermex PAT.P and CompACT AMS Ver. 3, Muromachi Kikai).

The detected jGCaMP7s signal was averaged within a 30 s session^19,39^. To detrend the gradual decrease of the signal during the recording days, the ± 12 h average from the time (145 points) was calculated as the baseline (F). The data were then detrended by subtracting F (ΔF). The ΔF/F value was then calculated. To determine the peak phase of the jGCaMP7s calcium signal, ΔF/F was smoothed with a 21-point moving average, and then the middle of the time points that crossed the value of 0 upward (Ca^2+^ onset) and downward (Ca^2+^ offset) were defined as peak phases (Fig. 4a). Additionally, the intervals between peak phases were defined as periods. A double-plotted actogram of the jGCaMP7s or EGFP signal was constructed by converting all ΔF to positive values by subtracting the minimum value of ΔF. These values were then multiplied by 100 or 1000 and rounded. The plots were made using ClockLab (Actimetrics) with normalization in each row. A double-plotted actogram of locomotor activity was also generated and superimposed on that of the jGCaMP7s signal.

The actogram of locomotor activity was used to determine the onset and offset of locomotor activity. Initially, we attempted to determine the onset and offset automatically, but this was followed by manual visual inspection and modifications by the experimenter^19,39^. To calculate the CT of the peak phases of the GCaMP signal, we defined the regression line of locomotor activity onsets as CT12.

We confirmed the jGCaMP7s expression and the position of the optical fiber by slicing the brains into 30 µm or 100 µm coronal sections using a cryostat (Leica). The sections were mounted on glass slides with a mounting medium (VECTASHIELD HardSet with DAPI, H-1500, Vector Laboratories) and observed with an epifluorescence microscope (KEYENCE, BZ-9000E).

### Supplementary Information

Supplementary Figures.

## Data Availability

All data reported in this paper will be shared by the corresponding author upon request.
