# Supplementary material for: In vivo recording of the circadian calcium rhythm in Prokineticin 2 neurons of the suprachiasmatic nucleus
Source: Sci Rep. 2023 Oct 9;13:16974. doi: 10.1038/s41598-023-44282-5 (PMC10562406; doi:10.1038/s41598-023-44282-5)
Supplement: Supplementary file 1 — Supplementary Figures. [file 41598_2023_44282_MOESM1_ESM.pdf]

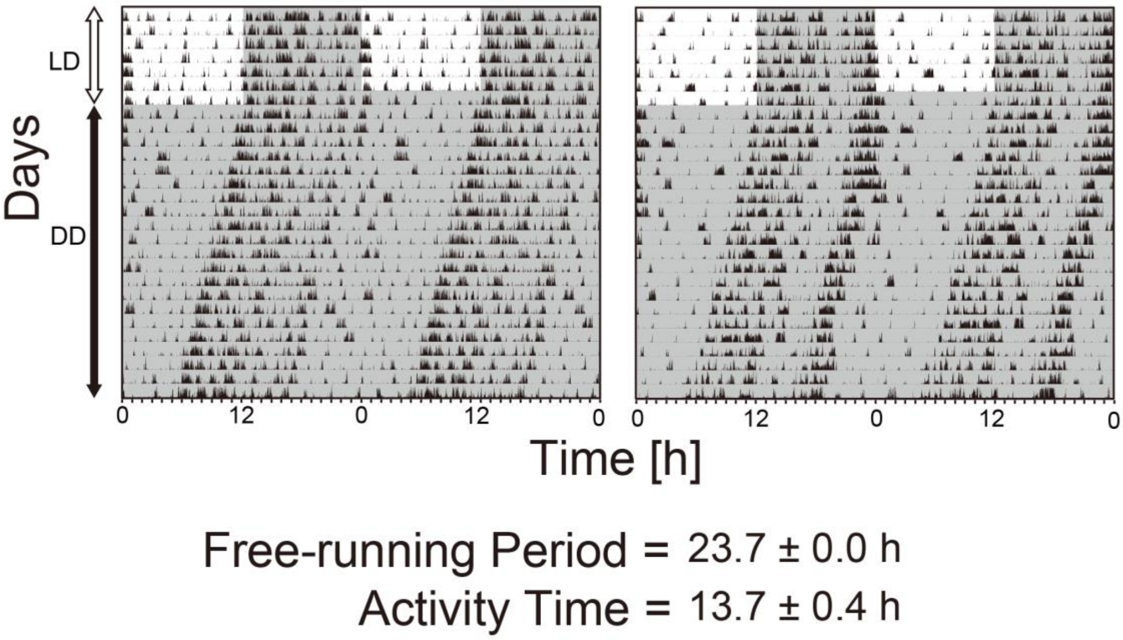

Supplementary Figure S1. Heterozygous *Prok2-tTA* mice show normal circadian behavior rhythm. Two representative actograms of the locomotor activity rhythm are shown. Animals were initially housed in LD conditions for 1 week and then transferred to DD for 3 weeks. Gray shadings in actograms indicate the dark periods. Free-running period and activity time were analyzed for the last 2 weeks in DD. The free-running period is 23.7 ± 0.0 h, and the activity time is 13.7 ± 0.4 h. Values are mean ± SEM. n = 4.

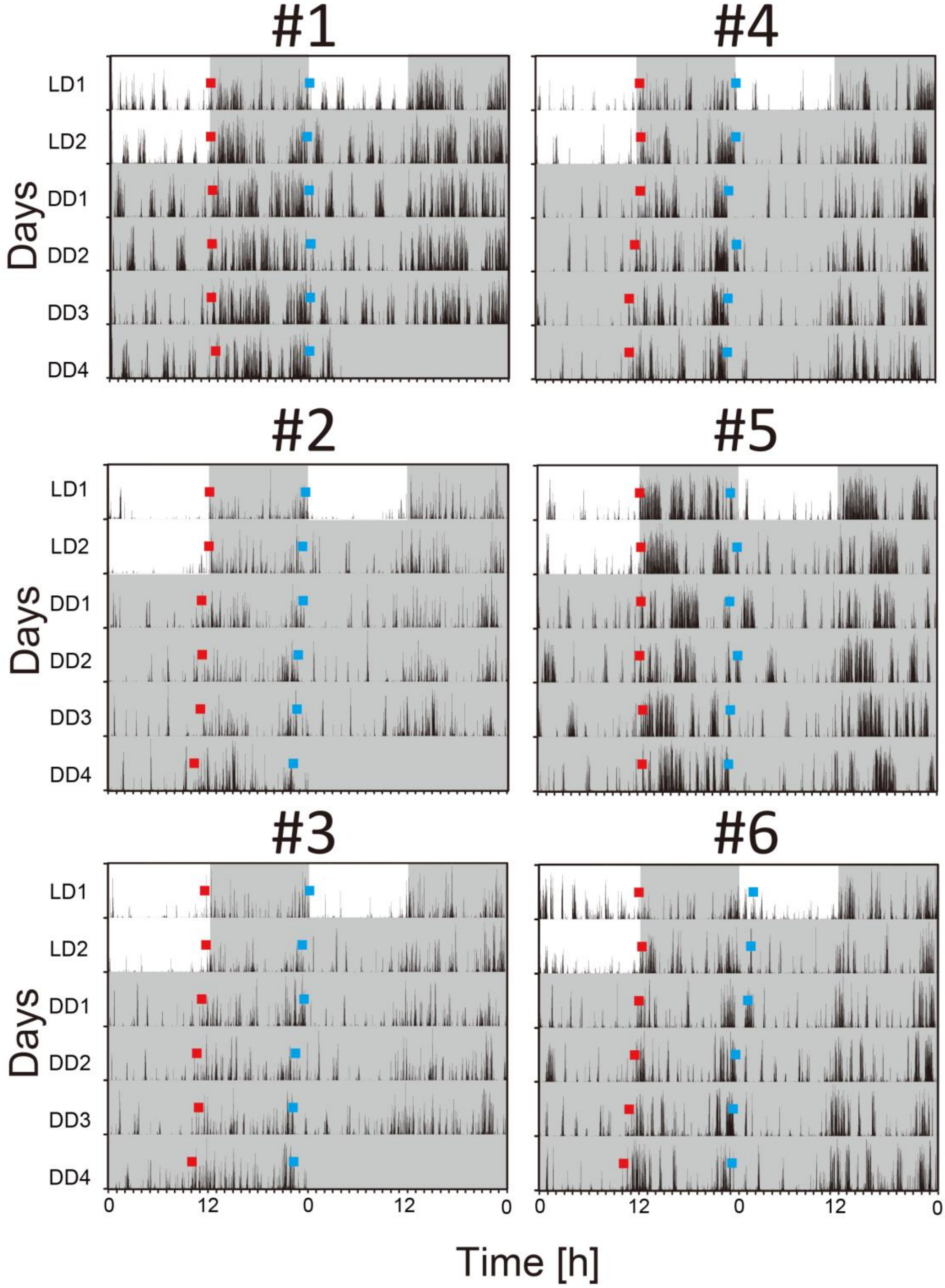

Supplementary Figure S2. Locomotor activity rhythms of individual mice recorded in vivo $[Ca^{2+}]_i$ rhythm in SCN Prok2 neurons. Mice were initially housed in LD (LD1, 2) and then in DD (DD1–4). The dark periods are represented as gray-shaded areas. The onset and offset of the locomotor activity, judged by visual inspection, are indicated by red and blue squares, respectively.
